# HIV knowledge and its correlation with the Undetectable = Untransmittable slogan in Brazil

**DOI:** 10.11606/s1518-8787.2022056004168

**Published:** 2022-09-30

**Authors:** Rayane C. Ferreira, Thiago Silva Torres, Luana Monteiro Spindola Marins, Maria das Graças B. Ceccato, Daniel R. B. Bezerra, Paula M. Luz

**Affiliations:** I Fundação Oswaldo Cruz Escola Nacional de Saúde Pública Sérgio Arouca Programa de Pós-graduação de Epidemiologia em Saúde Pública Rio de Janeiro RJ Brasil Fundação Oswaldo Cruz. Escola Nacional de Saúde Pública Sérgio Arouca. Programa de Pós-graduação de Epidemiologia em Saúde Pública. Rio de Janeiro, RJ, Brasil; II Fundação Oswaldo Cruz Instituto Nacional de Infectologia Evandro Chagas Laboratório de Pesquisa Clínica em DST e Aids Rio de Janeiro RJ Brasil Fundação Oswaldo Cruz. Instituto Nacional de Infectologia Evandro Chagas. Laboratório de Pesquisa Clínica em DST e Aids. Rio de Janeiro, RJ, Brasil; III Universidade Federal de Minas Gerais Faculdade de Farmácia Departamento de Farmácia Social Belo Horizonte MG Brasil Universidade Federal de Minas Gerais. Faculdade de Farmácia. Departamento de Farmácia Social. Belo Horizonte, MG, Brasil

**Keywords:** HIV Infections, transmission, HIV Seronegativity, Communicable Period, HIV Long-Term Survivors, Health Knowledge, Attitudes, Practice

## Abstract

Knowledge about HIV transmission and prevention is a necessary step for adopting preventive behaviors. We assessed HIV knowledge and its correlation with the perceived accuracy of the “Undetectable = Untransmittable” (U=U) slogan in an online sample with 401 adult Brazilians. Overall, 28% of participants showed high HIV knowledge level. The perceived accuracy of the U=U slogan significantly correlated with HIV knowledge. Younger participants, those reporting lower income or lower education, or who had never tested for HIV showed poorer HIV knowledge. Filling gaps of knowledge among specific populations is urgent in order to increase preventive behaviors and decrease HIV stigma.

## INTRODUCTION

Theoretical models of human behavior are proposed representations of how psychological constructs guide behavior^1^. The Health Belief Model, an example of such models, incorporates knowledge as a key component of behavior change^2^. In the case of HIV, models hypothesize that knowledge about HIV transmission can guide behavior, as well as the adoption of preventive strategies^3^. As such, knowledge may yield individual-level benefits for HIV-negative individuals as they adopt preventive behaviors^3^.

Greater HIV knowledge, especially of treatment as prevention, can decrease internalized HIV-related stigma and empower people living with HIV, to seek and adhere to antiretroviral treatment. Treatment as prevention, or antiretroviral treatment use and adherence, leading to HIV viral load suppression and, consequently, the prevention of HIV sexual transmission to HIV-negative partners^4,5^, has been disseminated beyond the scientific community with the slogan “Undetectable = Untransmittable” (U=U)^6^. A previous study shows that the perceived accuracy of the U=U slogan reduces internalized stigma or the experience of negative feelings or thoughts about oneself due to the HIV status^7^. At the population level, broad understanding of U=U and treatment as prevention can reduce stigma and discrimination against people living with HIV^7^.

Monitoring HIV knowledge informs about the level of understanding of how HIV is transmitted, on awareness about HIV prevention and on the degree of HIV stigma. Our study assessed HIV knowledge and its correlation with perceived accuracy of the U=U slogan in an online sample of Brazilians.

## METHODS

A convenience sample was recruited during October/2019 to complete an online survey through advertisements on social media (Facebook and WhatsApp) and Grindr, a geospatial network app used by sexual and gender minorities (SGM). Participant eligibility included age ≥ 18 years and residence in Brazil. Exclusion criteria were self-reported previous completion of the questionnaire and incorrect answers to attention questions (assuming that participants who erroneously answered these questions failed to pay attention to them)^8^. This study was approved by INI Evandro Chagas-FIOCRUZ institutional review board (#CAAE 01777918.0.0000.5262) in accordance with all applicable regulations. All study participants provided electronic informed consent before initiating the survey. Informed consent information included the objective of the study, time required for answering the survey, which and how data were stored, and investigators’ name and personal contact. No identifiable personal information was collected.

The survey instrument included items on socio-demographic information, prior HIV testing, HIV test results, the 12-item HIV/Aids Knowledge Assessment tool (HIV-KA)^9^, and a single question about the perceived accuracy of U=U, developed in English^10^ and translated to Brazilian Portuguese following standard protocols^11,12^. The HIV-KA was developed in Portuguese^9^, validated among Brazilian men who have sex with men (MSM)^9^, and recently administered among Brazilian MSM eligible for pre-exposure prophylaxis^13^. HIV-KA items (for example, “There are medications HIV-negative people can take to prevent HIV infection”) are described elsewhere^9,13^. Response options for all items were either “true”, “false” or “I do not know”, and the total score was calculated by summing all correct responses (established during scale development)^9^; “I don-t know” responses were deemed incorrect^9^. The perceived accuracy of the U=U slogan was assessed by the question: “With regards to HIV-positive individuals transmitting HIV by sexual contact, how accurate do you believe the U=U slogan is?” as used previously^11^. Response options were based on a Likert-type scale from “completely accurate” to “completely inaccurate” plus a fifth option (I do not know what “undetectable” means)^11^.

Participants’ characteristics were described by means, standard deviations (SD), and frequency distributions. HIV-KA scores (mean and SD) were estimated globally and by participants’ characteristics, including stratification by participants’ gender and sexual orientation (SGM [cisgender men who self-identified as gay/homosexual or bisexual, and transgender or non-binary individuals] *versus* other populations [cisgender men who self-identified as heterosexual and cisgender women]). Total HIV-KA scores were re-calculated to a 0 to 10 scale. The 25 and 75 percentiles of the HIV-KA scores were considered for categorizing knowledge levels into low (< 25^th^ percentile), medium (25^th^ to 75^th^ percentile), and high (> 75^th^ percentile). Finally, we used t- and chi-square tests to assess correlations between knowledge levels (mean score and knowledge level) and the perceived accuracy of the U=U slogan (completely accurate *versus* not [partially accurate/partially inaccurate/completely inaccurate]). Participants who reported not knowing what undetectable meant or for whom the response was missing were not considered in this analysis. All analyses were conducted in R Software, version 4.0.2, library epiDisplay (r-project.com).

## RESULTS

Overall, 401 participants completed the online questionnaire. Participants’ characteristics were: mean age 41.3 (SD 14.5) years, 60.3% were cisgender men, and 44% self-identified as gay/homosexual (Table). A quarter (25.3%) had never tested for HIV and, among those who reported their test results, 10.8% reported to be HIV-positive.

**Table:** Participants’ characteristics and knowledge scores as measured by the HIV/Aids Knowledge Assessment tool – HIV-KA (n = 401).

	n (%)	Knowledge scores
**Age**		
18–24	48 (12.0)	**8.2 (1.7)**
25–29	49 (12.2)	**9.1 (1.2)**
30–39	113 (28.2)	**8.9 (1.1)**
40–49	77 (19.2)	**8.7 (1.0)**
50–59	58 (14.5)	**8.3 (1.1)**
≥ 60	56 (14.0)	**7.4 (1.4)**
**Gender**		
Cisgender men	242 (60.3)	**8.8 (1.3)**
Cisgender women	150 (37.4)	**8.0 (1.3)**
Transgender or non-binary	9 (2.2)	**7.9 (1.1)**
**Sexual orientation**		
Gay/homosexual	175 (44.0)	**9.0 (1.2)**
Bisexual	53 (13.3)	**8.7 (1.2)**
Heterosexual	170 (42.7)	**7.9 (1.3)**
**Race**		
White	235 (59.8)	8.5 (1.3)
Black	41 (10.4)	8.6 (1.2)
Mixed	117 (29.8)	8.5 (1.4)
**Income level**		
Low	111 (27.7)	**8.3 (1.5)**
Middle	181 (45.1)	**8.5 (1.3)**
High	109 (27.2)	**8.7 (1.2)**
**Education**		
≤ 12 years	120 (30.2)	**8.1 (1.5)**
>12 years	278 (69.8)	**8.7 (1.1)**
**Region**		
North	7 (1.7)	8.3 (2.0)
Northeast	50 (12.5)	8.4 (1.2)
Midwest	30 (7.5)	8.5 (1.4)
Southeast	247 (61.6)	8.5 (1.4)
South	67 (16.7)	8.6 (1.3)
**Living in state capitals**		
No	132 (32.9)	**8.2 (1.4)**
Yes	269 (67.1)	**8.6 (1.3)**
**Ever tested for HIV**		
Never	99 (25.3)	**8.0 (1.5)**
< 6 months	131 (33.4)	**9.1 (1.1)**
> 6 months	162 (41.3)	**8.4 (1.2)**
**HIV status**^a^		
Negative	264 (89.2)	**8.6 (1.3)**
Positive	32 (10.8)	**9.6 (0.5)**
**Overal**l** HIV-KA score**	401 (100)	8.5 (1.3)
**Level of knowledge**^b^		
Low (< 25^th ^percentile)	42 (11.7)	NA
Medium (25^th^ to 75^th^ percentile)	217 (60.6)	NA
High (> 75^th^ percentile)	99 (27.7)	NA
**Perceived accuracy of U=U**		
Completely accurate	128 (31.9)	9.2 (1.0)
Accurate	69 (17.2)	9.0 (1.0)
Inaccurate	38 (9.5)	8.6 (1.2)
Completely inaccurate	123 (30.7)	7.8 (1.3)
Does not know what undetectable means	41 (10.2)	7.5 (1.4)
Missing	2 (0.5)	7.5 (1.2)

U=U: Undetectable = Untransmittable; NA: not applicable.

Note: bold indicates that differences in scores by characteristics were statistically significant (p < 0.05).

^a ^Six individuals failed to report their HIV status (n = 296).

^b^ Includes all responses except for “I do not know what undetectable means” or missing (n = 358).

We observed lower scores for the age groups in the extremes of our sample (18–24 and ≥ 50 years, p < 0.001), and higher scores among cisgender men, gay/homosexual, and those reporting higher income or education. Additionally, those reporting more recent HIV testing and an HIV-positive status showed higher scores. We noted significantly higher HIV knowledge among SGM: 40.4% (86/213) and 7.4% (14/188) showed high knowledge level among SGM and other populations, respectively (p < 0.001). The mean HIV-KA score was 8.5 (SD = 1.3); 27.7% of participants showed high knowledge level.

Mean HIV-KA score was higher among those who perceived U=U as completely accurate *versus* other (9.2 [SD = 1] *versus* 8.3 [SD = 1.3]; p < 0.001). Note that 44.5% of those who perceived U=U as completely accurate (n = 128) *versus* 18.3% of those who did not (n = 230) showed high knowledge levels (p < 0.001).

## DISCUSSION

Our results show that perceived accuracy of the U=U slogan was significantly correlated with HIV knowledge in Brazil, suggesting a converging understanding of HIV transmission and the implications of HIV treatment. People living with HIV showed higher knowledge than those who had recently tested themselves, as observed in a previous study^11^. HIV knowledge was also higher among SGM, but lower among young, lower-income, and lower-educated individuals. Compared to previous studies conducted in 2009 and 2016, our sample showed a higher knowledge level^9,14^.

Our results show that SGM scored higher in the HIV-KA measure, which may derive from SGM’s shared identity, including language and social context, which may facilitate communication and information sharing^15^. Moreover, HIV disproportionately impacts SGM and, as such, educational campaigns in the past decade have focused on disseminating information about HIV transmission and testing, and prevention services for this population. However, strategies are need to more broadly expand HIV knowledge, as well as U=U findings, to reduce HIV-related stigma and discrimination against people living with HIV in the population at large^7^. Advertisements on social media, mass media (e.g., TV, newspapers), consistent delivery of information about HIV and other sexually transmitted infections by health care providers, and sexual education programs at schools are of utmost importance to increase HIV knowledge among the general population.

We found that HIV knowledge varied significantly as a function of participants’ age, with higher knowledge in the intermediate age groups (25–49 years). These findings differ slightly from a prior Brazilian study in which those aged ≥ 25 years showed higher knowledge levels^9^. Similar to our findings, studies among MSM in the United Kingdom^16^ and USA^17^ found that lower knowledge levels were associated with the extremes in the age range. Taken together, these results suggest the need to increase HIV knowledge (e.g., social media advertisement) among younger populations, especially young MSM, a group whose vulnerability to HIV infection has increased during the recent years^18^.

Our results also show that knowledge scores differed by income and educational level, with those with higher income or education scoring significantly higher. In a recent study among SGM in the USA, racial minorities and lower-income individuals were less likely to have heard of the U=U slogan, with Latinxs showing higher odds of uncertainty about the slogan^19^. Community engagement could be beneficial in increasing knowledge among low-income and low-education SGM that may not be reached by online campaigns.

We also found that scores were significantly higher among those who had tested for HIV in the past, which may indicate that the act of testing offers an opportunity to gain information, counseling, and, ultimately, knowledge about HIV transmission, as previously suggested^17^. Conversely, the fact that an individual is searching a health service for HIV testing may indicate increased knowledge and awareness about HIV, including risk of transmission and its relation with sexual behavior. Nevertheless, healthcare providers must use this interaction with individuals at the time of HIV testing to increase HIV knowledge, including prevention strategies and the U=U slogan.

The strengths of our study include its diverse population, regarding gender identity, sexual orientation, and HIV status. Limitations include a convenience sample of individuals with access to the internet and social media, hindering the generalization of the findings to broader Brazilian populations, and self-reported data that may be subject to bias; though individuals might be more honest in online surveys, which tend to reduce social desirability biases.

In conclusion, our results suggest that the perceived accuracy of the U=U slogan was significantly correlated with HIV knowledge. Future studies are needed to understand and fill knowledge gaps among specific groups of the Brazilian population to decrease HIV transmission and stigma and, ultimately, end the HIV epidemic.
